# A comparative study of flexible ureteroscopic lithotripsy for upper urinary tract stones in patients with prior urosepsis following emergency drainage via retrograde ureteral stent or percutaneous nephrostomy

**DOI:** 10.1186/s12894-023-01369-5

**Published:** 2023-11-28

**Authors:** Sucai Liao, Xiang Xu, Yuan Yuan, Keiyui Tang, Genggeng Wei, Zhengquan Lu, Lin Xiong

**Affiliations:** 1 Department of Urology, The University of Hong Kong-Shenzhen Hospital, 1 Haiyuan First Road, Futian District, Shenzhen, Guangdong 518053 China; 2 Department of General Surgery, University Hospitals Plymouth NHS Trust, Derriford Hospital, Plymouth, UK

**Keywords:** Urosepsis, Retrograde ureteral stent, Percutaneous nephrostomy, Flexible ureteroscopic lithotripsy

## Abstract

**Background:**

Patients with urosepsis associated with upper urinary tract stones require further stone management after emergency drainage.

**Objective:**

To evaluate the safety and efficacy of elective flexible ureteroscopic lithotripsy (F-URSL) for upper urinary tract stones in patients with prior urosepsis who have undergone emergency drainage using retrograde ureteral stent(RUS) or percutaneous nephrostomy (PCN).

**Method:**

Between January 2017 and December 2021, clinical data were collected for 102 patients who underwent elective F-URSL following emergency drainage for urosepsis caused by upper ureteral or renal stones. The patients were categorized into two groups based on the drainage method used: the RUS group and the PCN group. The collected data included patient demographics, stone parameters, infection recovery after emergency drainage, and clinical outcomes post F-URSL. Subsequently, the data underwent statistical analysis.

**Results:**

A total of 102 patients were included in the statistical analysis, with 58 (56.86%) in the RUS group and 44 (43.14%) in the PCN group. Among the patients, 84 (82.35%) were female and 18 (17.65%) were male, with an average age of 59.36 years. Positive urine cultures were observed in 71 (69.61%) patients. Successful drainage was achieved in all patients in both groups, and there were no significant differences in the time required for normalization of white blood cell count (WBC) and body temperature following drainage. Additionally, all patients underwent F-URSL successfully, and no statistically significant differences were observed between the two groups in terms of operative time, stone-free rates, postoperative fever, and postoperative hospital stay.

**Conclusion:**

Both RUS and PCN have been established as effective approaches for managing urosepsis caused by upper urinary tract stones. Furthermore, the impact of these two drainage methods on the subsequent management of stones through elective F-URSL has shown consistent outcomes.

## Introduction

Sepsis is defined as a life-threatening organ dysfunction caused by a dysregulated host response to infection [1]. In 2017, the global incidence of sepsis was estimated at 48.9 million cases, resulting in 11 million sepsis-related deaths, accounting for 19.7% of global mortality [2]. Urosepsis in adults comprises approximately 25% of all sepsis cases and is primarily caused by obstructed uropathy in the upper urinary tract, such as ureteric stones [3, 4]. The primary treatments for urosepsis involve eliminating infectious foci and administering intravenous antibiotics [5]. Patients with urosepsis who do not receive surgical drainage are more than twice as likely to die compared to those who underwent drainage. Currently, percutaneous nephrostomy (PCN) and retrograde ureteral stent (RUS) are the main drainage methods [6]. There are varying opinions among scholars regarding the superiority of PCN versus RUS for urosepsis drainage. Some studies suggest that PCN may offer improved drainage outcomes with less patient discomfort [7, 8], while others report no significant difference between the two approaches [9, 10]. As there is no established guidance, the selection of drainage method often relies on the surgeon’s experience.

Urosepsis caused by upper urinary tract stones requires further stone management after sepsis treatment. Studies have demonstrated variations in surgical approaches for stone management based on the drainage method used. Patients who undergo RUS often proceed to F-URSL, while patients with PCN drainage tend to opt for percutaneous nephrolithotomy for stone treatment [8]. Therefore, the selection of the drainage method for urosepsis patients should take into account the subsequent stone management. However, there is a scarcity of comparative analyses assessing the effectiveness and safety of F-URSL in urosepsis patients who have underwent drainage via RUS or PCN. Some researchers claim that the presence of nephrostomy tubes (NT) in PCN drainage reduces the postoperative infection compared to RUS drainage followed by F-URSL. Nonetheless, there is insufficient evidence to support this claim. Therefore, this study aims to evaluate the safety and efficacy of elective F-URSL for upper urinary tract stones in patients with prior urosepsis who have underwent emergency drainage using RUS or PCN. Clinical data were collected between January 2017 and December 2021, involving 102 patients who underwent elective F-URSL following emergency drainage for urosepsis caused by proximal ureteral or kidney stones.

## Patients and methods

### Subjects

A retrospective analysis was conducted on 102 patients admitted to the Department of Urology at The University of Hong Kong-Shenzhen Hospital between January 2017 and December 2021. All patients had urosepsis due to proximal ureteral stones or kidney stones. The patients were divided into two groups based on the drainage method used: 58 patients in the RUS group and 44 patients in the PCN group. Diagnostic procedures included abdominopelvic computed tomography (CT), urinalysis, blood analysis, and urine bacterial culture, confirming the presence of kidney stones or proximal ureteral stones as well as the diagnosis of urosepsis. Sepsis diagnosis was based on established criteria: body temperature > 38 °C or < 36 °C, white blood cell count(WBC) > 12 × 10^9^/L or < 4 × 10^9^/L, and a quick Sequential Organ Failure Assessment (qSOFA) score of ≥ 2. The qSOFA score included criteria such as hypotension (systolic blood pressure ≤ 100mmHg), high respiratory rate (≥ 22 breaths/minute), and altered mental status (Glasgow Coma Score < 15), each scored as 1 point, resulting in a score ranging from 0 to 3 points [1]. Upon sepsis diagnosis, the patients received fluid resuscitation and anti-infective therapy, such as piperacillin sulbactam or meropenem, or therapy based on available bacterial cultures. Emergency drainage was performed using either RUS or PCN. Following recovery, all patients underwent elective F-URSL within 2–4 weeks.

### Drainage method

#### Retrograde ureteral stent (RUS)

RUS was performed with the patient in the lithotomy position under local or general anesthesia. The ureteroscope was placed through the urethra and the Zebra guidewire was inserted into the ureter through the ureteroscope. If the presence of visible pus-filled secretions at the ureteral orifice indicates the entry of the guidewire into the renal pelvis from beside the stone, an 5Fr D-J stent was placed along the guidewire. Intraoperative C-arm X-ray was performed to confirm the position of the D-J stent. If insertion fails, the ureteroscope was placed through the ureter below the stone, the guidewire was placed into the renal pelvis under direct vision through the ureteroscope, and the 5Fr D-J stent was inserted along the guidewire. Postoperative kidney-ureter-bladder radiography(KUB) was used to confirm the position of the D-J stent.

#### Percutaneous nephrostomy (PCN) PCN

was performed with the patient placed in the lateral position under local anesthesia. The procedure was performed utilizing CT guidance, an 18G puncture needle was percutaneously inserted into the intended renal calyces. After confirming urine outflow, a guidewire was inserted, and subsequently, an 7Fr nephrostomy tube was guided into the renal pelvis along the guidewire. The positioning of the NT was confirmed through postoperative KUB.

#### Flexible ureteroscopic lithotripsy(F-URSL)

The patients underwent elective F-URSL 2–4 weeks after infection control. Preoperative urine bacterial culture yielded negative results, and blood analysis showed normal leukocyte counts. Piperacillin-tazobactam was administered for perioperative prophylactic anti-infective treatment. The procedure was performed with the patient under general anesthesia in the lithotomy position. During the procedure, an 8-9.8Fr rigid ureteroscope was inserted into the ureter under direct vision. In the RUS group, the D-J tube was removed first. If the ureter was narrow, dilation was performed using an 18Fr balloon dilator. A guidewire was inserted through the ureteroscope up to the kidney, and a 12/14Fr ureteral access sheath(UAS) was placed along it. The flexible ureteroscope was then inserted through the sheath to locate the stones, and a 200 μm holmium laser fiber was used to fragment the stones. Irrigation during the procedure was performed using a 60ml syringe. After lithotripsy, a 5Fr D-J stent was inserted in all cases. In the PCN group, the NT was opened to reduce Intrapelvic pressure(IPP) during the procedure, and it was removed 24 h after the procedure. The D-J stent was removed 4 weeks later if no residual stones were present.

#### Data collection and analysis

The following information was collected and analyzed:(1) Preoperative clinical information: This included age, gender, BMI, stone size (in millimeters), stone laterality (right or left), location of stones (renal pelvis or proximal ureter), stone number (single or multiple stones), degree of hydronephrosis, urine culture results. (2) After emergency drainage: The time to normalize body temperature (in hours) and the time to normalize WBC count (in days) were recorded. (3) After F-URSL: The operation time of F-URSL (in minutes), stone-free status (defined as a residual stone size of < 4 mm by CT scan at 4 weeks after F-URSL), occurrence of postoperative fever (defined as a body temperature>38.0 °C), and postoperative hospital stay were analyzed.

### Statistical analysis

Continuous variables following a normal distribution were analyzed using t-tests, while those not conforming to a normal distribution were analyzed using the Mann-Whitney U-test. Categorical variables were compared using the chi-square test, and if any count was less than 5, the Fisher exact test was applied. Statistical significance was determined at a threshold of *P* < 0.05. Statistical analyses were performed using SPSS version 26.0 (IBM Corp., Armonk, NY, USA).

## Results

Patient demographics and variables are shown in Table 1. A total of 102 patients were included in the study, with 58 (56.86%) in the RUS group and 44 (43.14%) in the PCN group. The patient population consisted of 84 (82.35%) females and 18 (17.65%) males, with an mean age of 59.36 years. There was no significant differences were observed between the two groups in terms of age, gender, BMI, stone size, stone location, degree of hydronephrosis, history of diabetes mellitus, and other clinical characteristics. The urine bacterial culture results indicated positive cases in 71 patients, representing 69.61% of the total participants. Among them, 42patients (72.41% of the RUS group patients) were in the RUS group and 29patients (65.91% of the PCN group patients) in the PCN group, with some patients showing mixed bacterial infections. Escherichia coli was the most common bacterium, infecting a total of 47 patients, accounting for 66.20% of all positive urine cultures, including 28 in the RUS group and 19 in the PCN group. The Clinical outcomes after drainage and F-URS are shown in Table 2. Emergency drainage was successfully performed in all patients in both groups, with no significant difference observed in the time to normalize body temperature( 4.75, IQR 2.00-28.50 vs. 2.00, IQR 2.00-20.50, *P* = 0.123) and WBC count ( 1.00, IQR 1.00–2.00 vs. 1.00, 1.00–2.00, *P* = 0.757) post-operation between the two groups. All patients successfully underwent F-URSL, and no significant differences were observed between the two groups in terms of operation time (35.00, IQR 27.90–45.50 vs. 41.25, IQR 30.50–47.50, *P* = 0.461), postoperative stone-free rate (86.21% vs. 86.36%, *P* = 0.982), the rate of postoperative fever (12.07% vs. 4.55%, *P* = 0.293), and postoperative hospital stay (2.00, IQR 2.00–2.00 vs. 2.00, IQR 2.00–3.00).

**Table 1:** Patient demographics and variables

Variables		RUS	PCN	*P*-value
Total patients		58	44	
Age, year		58.84 ± 12.37	60.05 ± 13.15	0.638
Gender	Male	9(15.52%)	9(20.45%)	0.700
	Female	49(84.48%)	38(79.55%)	
BMI M(P25,P75)		22.48(20.10, 25.20)	22.13(20.25, 23.55)	0.385
Diabetes Mellitus	YES	14(24.14%)	10(22.73%)	0.868
	NO	44(75.86%)	34(77.27%)	
Location of stone	Renal pelvis	15(25.86%)	13(29.55%)	0.681
	Upper ureteral	43(74.14%)	31(70.45%)	
Stone laterality	Left	31(53.45%)	19(43.18%)	0.363
	Right	26(44.83%)	25(56.82%)	
	Bilateral	1(1.72%)	0	
Stone number	Single	37(63.79%)	27(61.36%)	0.802
	Multiple	21(36.21%)	17(38.64%)	
Stone size(mm) M(P25,P75)		8.00(8.00,11.00)	11.00 (8.00,12.5)	0.085
Degree of hydronephrosis	Mild	42(72.41%)	26(59.09%)	0.403
	Moderate	13(22.41%)	14(31.82%)	
	Severe	3 (5.18%)	4(9.09%)	
Positive urine culture	YES	42(72.41%)	29(65.91%)	0.479
	NO	16(27.59%)	15(34.09%)	

Data presented as Median (IQR 25–75), Mean ± SD and frequency (percentage)as appropriate

**Table 2:** Clinical outcomes after drainage and F-URSL

Variables			RUS	PCN	*P*-value
Post drainage	Time to normalize body temperature(hours)		4.75(2.00;28.50)	2.00(2.00;20.50)	0.123
	Time to normalize WBC(days)		1.00(1.00;2.00)	1.00(1.00;2.00)	0.757
Post F-URSL	Stone-free status	YES	50(86.21%)	38(86.36%)	0.982
		NO	8(13.79%)	6(13.64%)	
	Postoperative fever	YES	7(12.07%)	2(4.55%)	0.293
		NO	51(87.93%)	42(95.45%)	
	Operation time of F-URS(min)		35.00(27.90;45.50)	41.25(30.50;47.50)	0.461
	Postoperative hospital stay(day)		2.00 (2.00;2.00)	2.00 (2.00;3.00)	0.915

Data presented as Median (IQR 25–75) and frequency (percentage)as appropriate

## Discussion

Urosepsis is a urological emergency with various risk factors, including diabetes mellitus, immunosuppressant use, stones, and advanced age [5]. While urosepsis is more common in women and the prevalence is about twice as high in women as in men [11, 12]. In our study, we observed a significantly higher prevalence of urosepsis in women than in men, with a ratio of approximately 4:1, and some patients had concurrent diabetes mellitus. The mean age of patients in both groups was 59.36 years, and advanced age as a risk factor for urosepsis. Effective early goal-directed therapy for urosepsis involves broad-spectrum intravenous antibiotics, supportive treatment, and source control [13]. Surgical drainage of the infection source has been shown to reduce the mortality rate of urosepsis from 19.2 to 8.82% [6]. Stone is a common cause of urosepsis, and the current drainage methods for urosepsis caused by stones are RUS and PCN, but their superiority has been variable evaluations. In our analysis, we observed a rapid decrease in body temperature and WBC count to normal levels after emergency drainage, with no significant difference in the time required between the two drainage methods. Consequently, we concluded that there was no statistically significant difference in the effectiveness of the two drainage methods, consistent with the results reported by Ramsey and Pearle [9, 10].

During the performance of RUS for upper ureteral stones, especially in cases of multiple ureteral stones, it may be necessary to position the ureteroscope below the stone and insert a guidewire into the renal pelvis under direct vision to successfully place a D-J stent. Some prior studies have suggested that this approach could raise IPP, potentially worsening the infection [14]. However, other scholars argue that there is limited evidence to support this viewpoint [9]. In our study, as reviewed, we found no significant difference in the time to normalize body temperature and WBC count after drainage in both groups, indicating that drainage by RUS is safe. According to certain scholars, pre-stenting with a D-J stent in the ureter can enhance the success rate of UAS implantation and F-URSL [15]. In our study, certain patients encountered difficulties in placing the UAS during F-URSL due to ureteral stenosis, however, successful UAS placement was achieved after dilating the stenotic segment using a balloon dilator. All patients ultimately underwent successful F-URSL. The dilation of ureteral stricture segments using a ureteral balloon dilator has proven to enhance the success rate of UAS placement and reduce the need for secondary procedures [16].

Hydronephrosis resulting from urinary tract stones creates a conducive environment for bacterial growth and colonization, potentially leading to severe urosepsis [17]. Notably, most stones are heavily colonized with bacteria, with approximately half of the patients with positive stone cultures having negative bladder urine bacterial cultures [18]. Additionally, nearly a quarter of patients with positive preoperative urine cultures showed inconsistencies in the bacterial species between the stone and urine cultures [19]. Although preoperative treatment with sensitive antibiotics can effectively eradicate bacteria in the urine, it may not eradicate bacteria concealed within the stone matrix as antibiotics cannot penetrate it [20]. During F-URSL, fractured stones can release bacteria and endotoxins from the stone into the urine within the renal pelvis. When the IPP increases, the released bacteria and endotoxins can be carried back into the bloodstream along with the fluid, causing infection. Therefore, when managing stones with F-URSL in patients with prior urosepsis, reducing intraoperative IPP and minimizing the return of fluid to the renal pelvis, especially when complete elimination of bacteria from the stone is not possible, becomes crucial in minimizing postoperative infections.

In our study, both groups of patients who underwent F-URSL, the operative time and stone-free rate were similar, and there was no statistically significant difference in postoperative hospital stay and the rate of postoperative fever. This contrasts with the initial expectation that the incidence of postoperative fever would be lower in the PCN group than in the RUS group when F-URSL was performed after drainage due to the presence of a NT in the PCN group that could potentially reduce IPP. This outcome may be attributed to the use of a UAS during the procedure and the administration of longer and more potent antibiotics during the perioperative period. These factors likely contributed to the outcomes observed in our study. As all patients had prior urosepsis and indwelling D-J stent or NT, which were at increased risk of infectious complications after F-URSL [21–23]. However, there are no guidelines for perioperative antibiotic use in such cases. Therefore, to minimize the risk of infection, we referred to previous urine culture results and opted for potent antibiotics, such as piperacillin, as perioperative prophylaxis during F-URSL.

The physiological pressure in the renal pelvis typically ranges from 0 to 20 cmH_2_O [24]. During F-URSL, saline is infused continuously to maintain a clear surgical field, resulting in increased IPP. When IPP exceeds 27.2 cmH_2_O, fluid in the renal pelvis can return to the bloodstream via various pathways, including renal pelvic veins, renal tubules, and renal lymphatics [25]. Studies monitoring IPP during F-URSL revealed average values of 63 cmH_2_O when the endoscope was introduced into the kidney without a UAS, and 115.3 cmH_2_O during laser lithotripsy, with maximal irrigation pressures reaching 289.3-436.9 cmH_2_O [26]. High IPP is linked to postoperative fever, systemic inflammatory response syndrome (SIRS), and urosepsis [27–29]. The use of a UAS effectively reduces IPP. With the use of a UAS during F-URSL, IPP can be maintained below 30 cmH_2_O when the irrigation pressure is ≤ 100 cmH_2_O [30]. Research by Rehman et al. demonstrated the 12/14F access sheath provides for maximum flow of irrigant while maintaining a low intrarenal pelvic pressure. Even with an irrigation pressure of 200 cmH_2_O, renal pelvic pressure remained below 20 cmH_2_O [31]. Maintaining a low IPP depends not only on the size of the UAS, but also on the diameter of the flexible ureteroscope. When the ratio of the outer diameter of the flexible ureteroscope to the inner diameter of the UAS (Ratio of Endoscope-Sheath Diameter, RESD/REUS) is < 0.75, a low IPP can be maintained while ensuring adequate perfusion [32]. Therefore, we believe that during F-URSL, employing lower irrigation pressures, reduced flow rates, and an appropriately sized UAS can effectively maintain a lower pressure state in the renal pelvis, achieving similar effects as those of NT in lowering IPP, and reducing renal pelvic fluid reflux, subsequently reducing complications such as postoperative fever.

Our study demonstrated the effectiveness of both RUS and PCN in managing urosepsis due to stone obstruction. Additionally, we observed similar impacts of these drainage methods on subsequent F-URSL stone management. However, it is important to acknowledge the limitations of our study, being retrospective, single-center, and with a limited sample size, which might introduce bias. Therefore, future prospective studies with larger, multicenter populations are warranted to validate our findings more comprehensively.

## Conclusion

Both RUS and PCN have proven to be effective drainage methods for managing urosepsis due to stone obstruction. Furthermore, both drainage methods have a similar impact on the subsequent management of stones by F-URSL. Therefore, the selection of the drainage method should be based on the specific circumstances of the physician and hospital to optimize outcomes for patients with urosepsis and stone obstruction.

## Data Availability

The datasets used and/or analyzed during the current study are available from the corresponding author on reasonable request.

## Abbreviations

RUS: Retrograde ureteral stent
PCN: Percutaneous nephrostomy
F-URSL: Flexible ureteroscopic lithotripsy
WBC: White blood cell
SFR: Stone-free rate
UAS: Ureteral access sheath
IPP: Intrapelvic pressure
BMI: Body mass index
CT: Computed tomography
KUB: Kidney-ureter-bladder radiography
NT: Nephrostomy tube
